# Nephrotic Sydrome Developing in Severe Ovarian Hyperstimulation Syndrome

**Published:** 2013-12-22

**Authors:** Funda Gungor Ugurlucan, Burcin Karamustafaoglu, Ahmet Cem Iyibozkurt, Isin Kilicaslan, Yasar Caliskan, Mehmet Ozsurmeli, Ercan Bastu, Faruk Buyru

**Affiliations:** 1 Department of Obstetrics and Gynecology, Istanbul University, Istanbul Medical School, Istanbul, Turkey; 2 Department of Pathology, Istanbul University, Istanbul Medical School, Istanbul, Turkey; 3 Department of Internal Medicine, Division of Nephrology, Istanbul University, Istanbul Medical School, Istanbul, Turkey

**Keywords:** Ovarian Hyperstimulation Syndrome, In vitro Fertilization (IVF), Ovar- ian Stimulation, Ascites, Nephrotic Syndrome

## Abstract

We report a case that developed nephrotic syndrome during hospitalization for severe ovarian hyperstimulation syndrome without history of acute renal failure. During hospi- talization, she developed persistent ascites and respiratory distress. The 24 hours urine protein analysis revealed significant proteinuria and renal biopsy showed global and seg- mental sclerosis in glomeruli, mesangial arteritis, proliferations in visceral epithelial cells (IgA nephropathy). To the best of our knowledge, such a complication will be presented for the first time in the literature.

## Introduction

Ovarian hyperstimulation syndrome (OHSS) is one of the most dreadful complications of ovarian stimulation. Moderate to severe OHSS occurs in 0.2 to 2% of all ovarian stimulation cycles (1, 2). Risk factors for OHSS include young age, low body weight, polycystic ovarian syndrome, previous OHSS, pregnancy, high follicle count, elevated serum estradiol, using human chorionic gonadotropin (hCG) for luteal phase support, and rarely follicle stimulating hormone (FSH) mutations (3, 4). Despite extensive studies, there is still no such method for complete prevention of OHSS, except for ovulation triggering with gonadotropin-releasing- hormone analogues instead of hCG (5). Individualization of treatment, embryo freezing or single embryo transfer has the potential of reducing the risk and the severity of the syndrome in susceptible cases. However, still in various situations, those strategies may fail to prevent OHSS (6, 7).

 We report a case with nephrotic syndrome ensued during hospitalization for severe OHSS without history of acute renal failure. To the best of our knowledge, such a complication has not been identified in the literature.

## Case Report

A 34 years-old gravida 4 para 1 woman was admitted with abdominal distension, 6 days after embryo transfer. She had undergone controlled ovarian stimulation and in vitro fertilization (IVF); antagonist protocol with 150 IU recombinant FSH for 9 days, Cetrorelix for 5 days, and recombinant hCG were used for ovulation trigger. About 13 oocytes were retrieved and two embryos were transferred. She had a history of Caesarean section after a successful IVF cycle due to unexplained infertility 3 years ago without a history of OHSS. Transvaginal ultrasound showed ascites, enlarged ovaries (right ovary: 70×75 mm, left ovary: 80×85 mm) and endometrium appearing 10 mm in thickness. White blood cell count (WBC) and hemoglobin and hematocrit levels were 40900/L, 15.76 g/dl, and, 47.3%, respectively. Blood urea nitrogen (BUN), creatinine, total protein and albumin concentrations were 16 mg/dl, 1.0 mg/dl, 6.4 g/dl and, 3.3 g/dl, respectively. Urinalysis revealed no proteinuria. Body mass index (BMI) was 22 kg/m2. The patient was hospitalized with the diagnosis of severe OHSS, while intravenous fluid replacement, low molecular weight heparin, and vaginal progesterone were begun. Β-hCG was 6076 mIU/ml at hospitalization and decreased onwards. Chest tubes were inserted bilaterally due to severe dyspnea and bilateral pleural effusion, while paracentesis was performed several times due to abdominal distension. The biochemical values normalized during hospitalization in 2 months; however, ascites persisted and new-onset hypertension developed. The 24 hour urine protein analysis was 18.9 gr, while renal biopsy revealed global and segmental sclerosis in glomeruli and mesangial arteritis, as well as proliferations in visceral epithelial cells (IgA nephropathy, Figes1, 2). Corticosteroid and immunosuppressive treatment was begun, but symptoms of nephrotic syndrome persisted.

**Fig 1:**
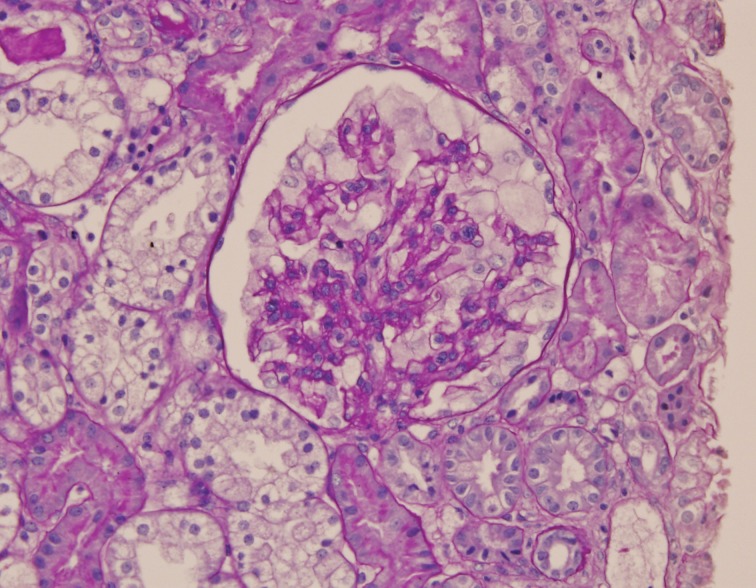
Glomerular mesangial proliferation, cytoplasmic foamy appearance and swelling of visceral epithelial cells.

**Fig 2:**
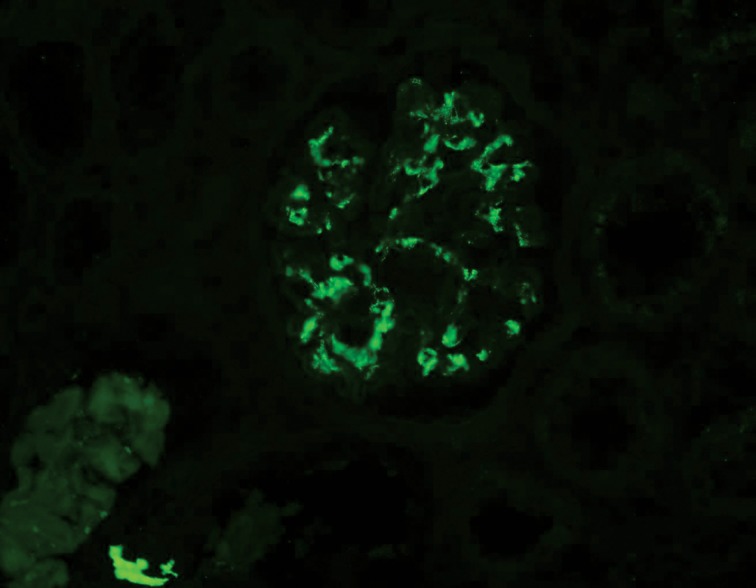
Mesangial granular IgA accumulation shown by immunofluorescence microscopy.

## Discussion

Ovarian hyperstimulation may develop as a result of controlled ovarian stimulation during assisted reproduction and is the most severe complication of the treatment (6, 8). The main features of OHSS are abdominal pain, nausea, vomiting, ascites, abdominal distention, localized or generalized peritonitis, acute abdominal pain, hypotension and/ or hypovolemia, dyspnea, electrolyte imbalance, and acute renal failure. The ultimate pathophysiological step underlying this clinical situation is increased vascular permeability. Secondary to administration of hCG for ovulation stimulation, the expression levels of vascular endothelial growth factor (VEGF) and VEGF receptor-2 (VEGFR-2) mRNA increase significantly leading to unwanted vascular permeability and edema. The treatment of OHSS is supportive including hospitalization in severe cases, administration of fluids, and thromboprophylaxis.

The renal complications, secondary to OHSS, include prerenal (hypovolemia) reasons, which is followed by the massive fluid shifts associated with increased vascular permeability (9). Additionally, in the literature, there are case reports of obstructive uropathies, secondary to distal ureter compression within the pelvis (10, 11). To the best of our knowledge, this is the first report of nephrotic syndrome developed as a complication of OHSS. This diagnosis was confirmed by 24-hour urine protein analysis and renal biopsy.

Nephrotic syndrome is characterized by heavy proteinuria (greater than 3.5 g/24 hours), hypoalbuminemia (less than 3.0 g/dL), and peripheral edema (12). Hyperlipidemia and thrombotic disease are also frequently observed (13, 14). IgA nephropathy is the most common lesion associated with primary glomerulonephritis (15). The major findings in light microscope analysis are focal or diffuse mesangial proliferation and matrix expansion (16). Ten percent of IgA nephropathy cases have acute presentation as nephrotic syndrome or acute glomerulonephritis characterized by edema, hypertension, hematuria, and renal insufficiency (17).

The history of our patient included an uncomplicated ovarian stimulation and pregnancy without OHSS. She developed proteinuria during the course of treatment of OHSS. She had persistent ascites, and also received corticosteroid and immunosuppressive treatment. Zhao et al. (18) reported a case of acute renal failure in a nephrotic patient with 4 days of treatment with hydroxyethyl starch (HES). Renal biopsy demonstrated mesangioproliferative glomerulonephritis with tubulointerstitial changes resembling acute tubulointerstitial nephritis. They suggested that an immune disease due to a hapten, induced by HES, may be a possible factor in the pathogenesis of acute renal failure. Our patient received hydroxyethyl starch for OHSS, but she did not develop acute renal failure during the course of treatment.

In conclusion, we present a case of nephrotic syndrome associated with severe OHSS with an unknown mechanism. In cases of severe OHSS with persistent symptoms and ascites resistant to treatment, 24 hour urine protein analysis may be useful. Reports from different centers and their experiences are warranted in order to define underlying mechanisms and to establish a prevention and treatment strategy for the particular complication.
